# Analysis of Neonatal Neurobehavior and Developmental Outcomes Among Preterm Infants

**DOI:** 10.1001/jamanetworkopen.2022.22249

**Published:** 2022-07-18

**Authors:** Elisabeth C. McGowan, Julie A. Hofheimer, T. Michael O’Shea, Howard Kilbride, Brian S. Carter, Jennifer Check, Jennifer Helderman, Charles R. Neal, Steve Pastyrnak, Lynne M. Smith, Marie Camerota, Lynne M. Dansereau, Sheri A. Della Grotta, Barry M. Lester

**Affiliations:** 1 Department of Pediatrics, Brown Alpert Medical School and Women and Infants Hospital, Providence, Rhode Island; 2 Department of Pediatrics, University of North Carolina School of Medicine, Chapel Hill; 3 Department of Pediatrics-Neonatology, Children’s Mercy Hospital, Kansas City, Missouri; 4 Department of Pediatrics, Wake Forest School of Medicine, Winston Salem, North Carolina; 5 Department of Pediatrics, University of Hawaii John A. Burns School of Medicine, Honolulu; 6 Department of Pediatrics, Spectrum Health–Helen DeVos Hospital, Grand Rapids, Michigan; 7 Department of Pediatrics, Harbor-UCLA Medical Center, Torrance, California; 8 Brown Center for the Study of Children at Risk, Brown Alpert Medical School and Women and Infants Hospital, Providence, Rhode Island; 9 Department of Psychiatry and Human Behavior, Brown Alpert Medical School, Providence, Rhode Island

## Abstract

**Importance:**

The ability to identify poor outcomes and treatable risk factors among very preterm infants remains challenging; improving early risk detection and intervention targets to potentially address developmental and behavioral delays is needed.

**Objective:**

To determine associations between neonatal neurobehavior using the Neonatal Intensive Care Unit (NICU) Network Neurobehavioral Scale (NNNS), neonatal medical risk, and 2-year outcomes.

**Design, Setting, and Participants:**

This multicenter cohort enrolled infants born at less than 30 weeks’ gestation at 9 US university–affiliated NICUs. Enrollment was conducted from April 2014 to June 2016 with 2-year adjusted age follow-up assessment. Data were analyzed from December 2019 to January 2022.

**Exposures:**

Adverse medical and psychosocial conditions; neurobehavior.

**Main Outcomes and Measures:**

Bayley Scales of Infant and Toddler Development, third edition (Bayley-III), cognitive, language, and motor scores of less than 85 and Child Behavior Checklist (CBCL) T scores greater than 63. NNNS examinations were completed the week of NICU discharge, and 6 profiles of neurobehavior were identified by latent profile analysis. Generalized estimating equations tested associations among NNNS profiles, neonatal medical risk, and 2-year outcomes while adjusting for site, maternal socioeconomic and demographic factors, maternal psychopathology, and infant sex.

**Results:**

A total of 679 enrolled infants had medical and NNNS data; 2-year follow-up data were available for 479 mothers and 556 infants (mean [SD] postmenstrual age at birth, 27.0 [1.9] weeks; 255 [45.9%] female). Overall, 268 mothers (55.9%) were of minority race and ethnicity, and 127 (26.6%) lived in single-parent households. The most common neonatal medical morbidity was BPD (287 [51.7%]). Two NNNS behavior profiles, including 157 infants, were considered high behavioral risk. Infants with at least 2 medical morbidities (n = 123) were considered high medical risk. Infants with high behavioral and high medical risk were 4 times more likely to have Bayley-III motor scores less than 85 compared with those with low behavioral and low medical risk (adjusted relative risk [aRR], 4.1; 95% CI, 2.9-5.1). Infants with high behavioral and high medical risk also had increased risk for cognitive scores less than 85 (aRR, 2.7; 95% CI, 1.8-3.4). Only infants with high behavioral and low medical risk were in the clinical range for CBCL internalizing and total problem scores (internalizing: aRR, 2.3; 95% CI, 1.1-4.5; total: aRR, 2.5; 95% CI, 1.2-4.4).

**Conclusions and Relevance:**

In this study, high-risk neonatal neurobehavioral patterns at NICU discharge were associated with adverse cognitive, motor, and behavioral outcomes at 2 years. Used in conjunction with medical risk, neonatal neurobehavioral assessments could enhance identification of infants at highest risk for delay and offer opportunities to provide early, targeted therapies.

## Key Points

**Question:**

Are neonatal neurobehavioral patterns associated with behavioral and neurodevelopmental impairment at age 2 years?

**Findings:**

In this cohort study of 556 infants born before 30 weeks’ gestation, infants with high-risk neurobehavioral profiles at neonatal intensive care unit discharge and varying medical complications were 4 times more likely to have motor delay, nearly 3 times more likely to have cognitive delay, and 2 times more likely to have behavioral concerns at age 2 years.

**Meaning:**

In this study, neurobehavioral assessments of preterm infants were useful for identification of those at risk for adverse outcomes beyond medical risk alone and could potentially lead to early, targeted interventions.

## Teaser

This cohort study evaluates associations between neonatal neurobehavior using the Neonatal Intensive Care Unit Network Neurobehavioral Scale (NNNS), neonatal medical risk, and 2-year outcomes.

## Introduction

Very preterm infants are vulnerable to a variety of medical complications while in the neonatal intensive care unit (NICU) that may compromise neurodevelopmental progression. While rates of morbidities may vary,^1,2,3^ they are associated with a spectrum of developmental outcomes, including cognitive, language, and motor.^4,5,6^ Behavior problems, reported in 13% to 46% of very preterm and low birth weight infants,^7^ are also of clinical concern, as early dysregulated behavior may affect long-term academic, home, and social functioning.^8^ However, challenges remain in refining our ability to identify poor developmental outcomes, in particular early behavioral problems.

Difficulties with regulation of attention, movement, arousal, and stress in high-risk groups may present as early as the neonatal period and can be captured via neurodevelopmental assessment, including neurobehavioral profiles on the NICU Network Neurobehavioral Scale (NNNS).^9^ The NNNS is a comprehensive bedside evaluation that incorporates a neurologic examination, behavioral measures, and signs of stress and has demonstrated long-term predictive value among high-risk preterm infants.^10,11,12^ NNNS profiles have been previously reported for the Neurobehavioral Outcomes of Very Preterm Infants (NOVI) cohort, and at-risk profiles have been associated with maternal prenatal demographic risk, infant risks of small birth head circumference and sepsis as well as differences in neonatal epigenetic patterns.^13,14,15,16^

If neonatal neurobehavioral assessments are early markers of neurologic integrity and function, then identifying high-risk patterns of neurobehavior may allow clinicians to better distinguish, prior to hospital discharge, those at greatest risk for later impairment. The overarching goal and primary NOVI study outcome was to determine associations between neonatal neurobehavior, medical risk, and neurodevelopmental and behavioral outcomes at 2 years. Our hypothesis was that neurobehavioral patterns will identify infants with neurodevelopmental and behavioral impairment on the Bayley Scales of Infant and Toddler Development, third edition (Bayley-III), and the Child Behavior Checklist (CBCL) beyond those identified by medical risk alone, thus supporting the novel role of neonatal neurobehavior in the prediction of outcome.

## Methods

Infants were enrolled in the multicenter NOVI study from April 2014 through June 2016 at 9 US university affiliated NICUs. Inclusion criteria were: (1) birth at less than 30 weeks’ gestation, (2) parental ability to read and speak English or Spanish, and (3) residence within 3 hours of the NICU and follow-up clinic. Exclusion criteria included maternal cognitive impairment (inability to provide informed consent), maternal age younger than 18 years, maternal or infant death, and infants with major congenital anomalies. Enrollment and consent procedures were approved by local institutional review boards. All mothers provided written informed consent. This study followed the Strengthening the Reporting of Observational Studies in Epidemiology (STROBE) guidelines for observational studies.

Infant neurobehavior was assessed using the NNNS, a standardized examination with established validity and reliability that has been used in a variety of high-risk infant populations.^9,17,18,19^ It includes measures of tone, reflexes, social and behavioral functioning, and stress signs. During the week of NICU discharge, this 15- to 20-minute examination was administered by certified NNNS examiners blinded to clinical course. Scores on individual items were converted to summary scores reflecting attention, handling, self-regulation, arousal, excitability, lethargy, hypertonia, hypotonia, nonoptimal reflexes, asymmetric reflexes, quality of movement, and stress abstinence.^17^

Procedures and criteria for recording maternal and infant variables have been previously described, including cranial ultrasonographic readings, interpreted by centralized study neuroradiologists.^13^ Infant medical risk, selected a priori, included 4 morbidities: brain injury (defined as periventricular leukomalacia, moderate to severe ventriculomegaly, or parenchymal echodensity), bronchopulmonary dysplasia (BPD), severe retinopathy of prematurity, and necrotizing enterocolitis and/or culture-positive sepsis.^20^

The main outcome measures were completed at 2 years’ adjusted age. Assessments included the Bayley-III, which was administered by licensed site practitioners, blinded to NICU and NNNS outcomes and trained by certified Bayley-III trainers to reliability using standardized Bayley-III protocols.^21^ Cognitive, language, and motor composite scores were derived (mean [SD] scores of 100 [15]), and scores less than 85 represent delays.^21,22,23^ Parents completed the CBCL, a self-report questionnaire consisting of 100 statements about the child’s behavior and responses recorded on a scale of 0 (not true), 1 (somewhat or sometimes true), or 2 (very true or often true). The CBCL internalizing problems score (composed of 4 syndrome scales: emotionally reactive, anxious and/or depressed, somatic complaints, and withdrawn behavior), externalizing problems score (composed of 2 syndrome scales: attention problems and aggressive behavior), and a total problems score were derived from summary scores of respective syndrome scales. Internalizing, externalizing, and total problems T scores greater than 63 were classified as clinically significant.^24^

### Statistical Analysis

#### NNNS Neurobehavioral Profiles

Latent profile analysis (LPA), using Mplus version 8.1 (Muthén and Muthén), of the NNNS summary scores was used to group infants into mutually exclusive categories that represent heterogeneous subgroups and have been previously reported.^13,14^ LPA models were fit with different numbers of profiles, and the model containing the optimal number of profiles was identified using model fit criteria. These include bayesian information criteria, the bootstrapped likelihood ratio test, and the number of cases in each profile. In the NOVI cohort, a 6-profile solution was identified (Table 1; eFigure in the Supplement). Infants in profiles 5 and 6 had scores that reflected the poorest functioning in multiple domains. Infants in profile 5 had low attention, low arousal with more lethargy and hypotonia, and the most nonoptimal reflexes compared with infants in the other profiles. Infants in profile 6 also had low attention, poor movement quality, and poor self-regulation, with elevated arousal, excitability, and hypertonia and more stress abstinence signs.

**Table 1.** NNNS Profiles *z* Scores^a^

NNNS summary score	Profile 1 (n = 79 [11.6%])	Profile 2 (n = 209 [30.7%])	Profile 3 (n = 78 [11.5%])	Profile 4 (n = 108 [15.9%])	Profile 5 (n = 158 [23.3%])^b^	Profile 6 (n = 47 [6.9%])^b^
Attention	1.33	−0.17	−0.26	0.49	−0.49	−0.48
Handling	−0.17	−0.51	0.79	0.54	−0.22	0.72
Regulation	1.32	0.23	−0.79	0.58	−0.50	−1.55
Arousal	−0.88	−0.30	1.34	0.27	−0.40	1.29
Excitability	−0.53	−0.64	1.13	0.01	−0.14	2.32
Lethargy	−0.32	0.14	−0.47	−0.64	0.74	−0.35
Hypertonicity	−0.08	−0.14	0.43	−0.16	−0.14	0.85
Hypotonicity	−0.34	−0.16	−0.14	−0.32	0.67	−0.02
Nonoptimal reflexes	−0.55	−0.24	−0.26	−0.63	1.05	0.35
Asymmetric reflexes	0.57	−0.43	−0.53	0.82	−0.04	0.07
Quality of movement	0.75	0.43	0.38	−0.08	−0.57	−1.69
Stress abstinence	−0.30	−0.66	0.16	0.51	0.25	1.16

Abbreviation: NNNS, Neonatal Intensive Care Unit Network Neurobehavioral Scale.

^a^ Infants with profile 1 had scores that reflect sustained, focused attention and well-modulated arousal tone, movement quality, and self-regulation; they required average handling assistance to sustain alertness. Infants with profile 2 had the fewest stress indicators and predominantly average performance on remaining scores. Infants with profile 3 required more handling assistance and had less self-regulation, with higher arousal and excitability. Infants with profile 4 needed increased handling assistance but showed modulated attention, tone, and self-regulation. Infants with profile 5 (a high behavioral risk profile) showed poorly sustained attention, low arousal with the most lethargy and hypotonia, and increased numbers of nonoptimal reflexes. Infants with profile 6 (a high behavioral risk profile) showed poorly sustained attention, self-regulation, and quality of movement in addition to extremely high levels of arousal, excitability, hypertonicity, and stress signs.

^b^ High behavioral risk profiles.

#### Medical Risk and Behavioral Risk

Medical and behavioral risk were dichotomized for statistical analyses. Infants were grouped by high medical risk (defined as ≥2 medical morbidities) vs low medical risk (≤1 medical morbidity) as well as high behavioral risk (defined as NNNS profiles 5-6) vs low behavioral risk (NNNS profiles 1-4). Medical risk of at least 2 morbidities in this sample of infants born less than 30 weeks’ postmenstrual age (PMA) is based on prior work^25^ and clinical recognition that the presence of at least 1 morbidity in this high risk group is likely. Profiles 5 and 6 were chosen as they reflected the poorest behavioral functioning.

Maternal and infant characteristics were summarized by medical risk and NNNS neurobehavioral profiles using frequencies for categorical characteristics and means for continuous characteristics. Three indicator variables were developed to determine the association of medical and behavioral risk with each developmental outcome: (1) low behavioral risk and high medical risk, (2) high behavioral risk and low medical risk, and (3) high behavior risk and high medical risk. The referent group was infants with low behavioral and low medical risk. Associations of behavioral and medical risk with 2-year outcomes were assessed using generalized estimating equations that accounted for a binomial outcome distribution and nesting of multiple births within families. Generalized estimating equations using a log link function to obtain beta weights that can be converted to relative risks (RRs) directly produced model convergence issues. We converted odds ratios from the generalized estimating equation models to RR by adjusting for disease prevalence.^26^

Covariates, selected a priori, included study site, maternal socioeconomic status (SES; defined as Hollingshead level V^27^), maternal race (minority racial or ethnic group [American Indian, Asian, Black, Hispanic, Native Hawaiian, Pacific Islander, or “other”] or White, specified by mother) primary language, partner status, infant gestational age at birth, and infant sex. Maternal psychologic risk was assessed using the Brief Symptom Inventory (BSI),^28^ which captures psychological distress. The BSI Global Severity Index (GSI) measures distress intensity and was averaged to cover the entire study period (discharge and 2 years). The GSI is the most sensitive indicator of the respondent’s stress level and indicates distress intensity. There are a total of 53 items on the BSI. The sums for the 9 symptom dimensions and additional items are added together and divided by the total number of responses (53 when there are no missing items). In a normative sample of adults the mean (SD) GSI was 0.30 (0.31). Higher scores indicate more distress intensity. Data were analyzed using SPSS version 24.0 (IBM Corp). Statistical significance was set at *P* < .05, and all tests were 2-tailed.

## Results

This study enrolled 704 infants, of whom 679 had medical data and NNNS assessments by NICU discharge (Figure). Follow-up data at age 2 years were available for 479 of 601 mothers (79.7%) and 556 of 679 infants (81.9%) (Table 2). The mean (SD) PMA was 27.0 (1.9) weeks, and 255 infants (45.9%) were female. Overall, 268 mothers (55.9%) were of minority race and ethnicity, and 127 (26.6%) were single-parent households. The most common neonatal medical morbidity was BPD (287 [51.2%]). There were no differences by maternal or infant characteristics for the 148 infants lost to follow-up. Of the 556 infants available for this study, 544 had both NNNS and medical data. There were 157 infants (28.9%) classified as high behavioral risk and 123 (22.6%) as high medical risk, with 44 (8.1%) classified as both high behavioral and high medical risk. Among infants with complete medical data and NNNS assessments, 519 infants had a complete Bayley-III, and 544 had a complete CBCL. Behavioral and medical risk groups differed by maternal primary language, SES, PMA at birth, BPD, brain injury, and necrotizing enterocolitis and/or sepsis (Table 3). Behavioral and medical risk groups also differed by Bayley-III and CBCL scores (eTable in the Supplement). The proportion of infants in the high behavioral and high medical risk group with Bayley-III cognitive, language, and motor scores less than 85 were 52.6% (20 infants), 55.3% (21 infants), and 52.6% (20 infants), respectively. Six infants (13.6%) in the high behavioral and high medical risk group had CBCL internalizing and total problem T scores greater than 63.

**Figure.**
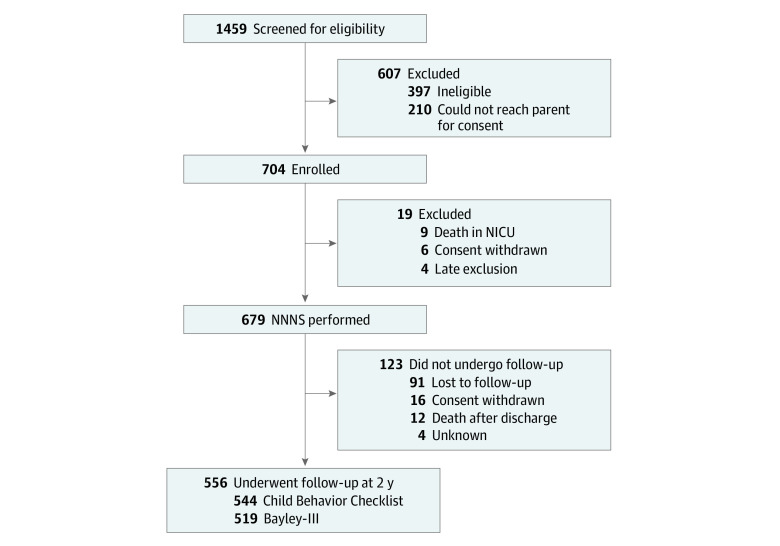
Study Flowchart Bayley-III indicates Bayley Scales of Infant and Toddler Development, third edition; NICU, neonatal intensive care unit; NNNS, NICU Network Neurobehavioral Scale.

**Table 2.** Maternal and Neonatal Characteristics of Dyads Included at the 2-Year Follow-up vs Children Lost to Follow-up^a^

Characteristic	No. (%)	
	Included	Lost to follow-up
**Maternal**		
No.	479	122
Minority race or ethnicity^b^	268 (55.9)	79 (64.8)
Low SES (Hollingshead level V)^c^	47 (9.8)	12 (9.9)
Single-parent household	127 (26.6)	25 (20.5)
Brief Symptom Inventory, average total from discharge and 2 y, mean (SD)	0.28 (0.30)	0.19 (0.18)
Multiple gestation	80 (16.7)	17 (14)
**Infant **		
No.	556	148
PMA at birth, mean (SD), wk	27.0 (1.9)	27.1 (1.9)
Sex		
Female	255 (45.9)	54 (38.0)
Male	301 (54.1)	94 (62.0)
Average medical risk, mean (SD)^d^	0.88 (0.87)	0.61 (0.96)
BPD^e^	287 (51.7)	70 (49.3)
Brain injury (PED, PVL, VDIL)^f^	68 (12.3)	24 (17.1)
NEC/sepsis^g^	103 (18.6)	25 (17.6)
Severe retinopathy of prematurity^h^	31 (5.6)	10 (7.0)

Abbreviations: BPD, bronchopulmonary dysplasia; NEC, necrotizing enterocolitis; PED, parenchymal echodensity; PMA, postmenstrual age; PVL, periventricular leukomalacia; SES, socioeconomic status; VDIL, ventricular dilation.

^a^ Maternal and infant characteristics did not differ statistically by included vs lost to follow-up groups.

^b^ Minority racial or ethnic group included American Indian, Asian, Black, Hispanic, Native Hawaiian, Pacific Islander, or “other,” as specified by the mother.

^c^ Socioeconomic status was calculated using the 4-factor Hollingshead Index (based on marital status, employment, education attainment, and occupation), which has been adapted to single-parent and nonnuclear families, with Hollingshead level V indicating low SES.

^d^ Count of medical risks, including BPD, brain injury, NEC/sepsis, and severe retinopathy of prematurity.

^e^ BPD defined as oxygen requirement at 36 weeks’ PMA.

^f^ Brain injury measured by most severe finding on any cranial ultrasonograph.

^g^ NEC/sepsis defined as Bells classification of stage 2 or greater.

^h^ Severe retinopathy of prematurity defined as stage 4 or 5 or requiring surgery.

**Table 3.** Maternal and Neonatal Characteristics at Birth by Medical Risk and Behavioral Risk^a^^,^^b^

Characteristic	Participants, No. (%)				*P* value
	Low behavioral risk with low medical risk	Low behavioral risk with high medical risk	High behavioral risk with low medical risk	High behavioral risk with high medical risk	
**Maternal characteristics**					
No.	263	70	97	39	
Minority race or ethnicity^c^	143 (54.4)	37 (52.9)	53 (54.6)	27 (69.2)	.34
Non-English primary language	42 (16.0)	12 (17.1)	25 (26.0)	12 (30.8)	.04
Low SES (Hollingshead category V)	18 (6.8)	4 (5.8)	15 (15.5)	8 (20.5)	.006
No partner	68 (25.9)	19 (27.5)	23 (23.7)	13 (33.3)	.70
Brief Symptom Inventory, average total from discharge and 2 y, mean (SD)	0.27 (0.28)	0.30 (0.31)	0.28 (0.32)	0.34 (0.42)	.57
**Infant characteristics**					
No.	308	79	113	44	
PMA at birth, mean (SD), wk	27.4 (1.8)	25.7 (1.8)	27.4 (1.8)	25.2 (1.8)	<.001
Female sex	149 (48.4)	31 (39.2)	48 (42.5)	19 (43.2)	.43
BPD	123 (39.9)	77 (97.5)	41 (36.3)	39 (88.6)	<.001
Brain injury (PED, PVL, VDIL)	9 (2.9)	31 (39.2)	6 (5.3)	20 (45.5)	<.001
NEC/sepsis	15 (4.9)	46 (58.2)	8 (7.1)	28 (63.6)	<.001
Severe retinopathy of prematurity	0	21 (26.6)	2 (1.8)	8 (18.2)	NA

Abbreviations: BPD, bronchopulmonary dysplasia; NA, not applicable; NEC, necrotizing enterocolitis; PED, parenchymal echodensity; PMA, postmenstrual age; PVL, periventricular leukomalacia; SES, socioeconomic status; VDIL, ventricular dilation.

^a^ Medical risks include the following neonatal morbidities: chronic lung disease, brain injury (PED, PVL, VDIL), NEC/sepsis, and severe retinopathy of prematurity. Low medical risk defined as no more than 1 morbidity; high medical risk defined as at least 2 morbidities.

^b^ Behavioral risk grouped by Neonatal Intensive Care Unit Network Neurobehavioral Scale profiles: low behavioral risk defined as profiles 1 to 4; high behavioral risk defined as profile 5 or 6.

^c^ Minority racial or ethnic group included American Indian, Asian, Black, Hispanic, Native Hawaiian, Pacific Islander, or “other,” as specified by the mother.

In unadjusted and adjusted models (Table 4), all 3 behavioral and medical risk groups were associated with motor scores less than 85; for infants with low behavioral and high medical risk, the RR was 2.8 (95% CI, 1.8-3.9), while those with high behavioral and low medical risk had an RR of 1.8 (95% CI, 1.1-2.7). Notably, for infants with high behavioral and high medical risk, the risk for motor delay was 4 times greater than that for the low behavioral and low medical risk group (adjusted RR [aRR], 4.1; 95% CI, 2.9-5.1). Additionally, among infants with high behavioral and high medical risk, cognitive delay was more likely (aRR, 2.7; 95% CI, 1.8-3.4). No associations in the adjusted model were identified between behavioral or medical risk and Bayley-III language scores. Infants in the high behavioral risk and low medical risk group were the only ones with increased risk of clinical range CBCL internalizing and total problems (internalizing: aRR, 2.3; 95% CI, 1.1-4.5; total: aRR, 2.5; 95% CI, 1.2-4.4).

**Table 4.** Two-Year Neurodevelopmental and Behavioral Outcomes Multivariable Models

Outcome	No.	Low behavioral risk with high medical risk		High behavioral risk with low medical risk		High behavioral risk with high medical risk	
		RR (95% CI)	aRR (95% CI)^a^	RR (95% CI)	aRR (95% CI)^a^	RR (95% CI)	aRR (95% CI)^a^
**Bayley-III**							
No.	220	76	76	106	106	38	38
Cognitive composite <85	131	1.4 (0.9-2.2)	1.2 (0.7-1.8)	1.1 (0.7-1.8)	1.2 (0.8-1.8)	2.6 (1.8-3.8)	2.7 (1.8-3.4)
Language composite <85	204	1.4 (1.1-1.9)	1.3 (0.9-1.7)	1.1 (0.8-1.5)	1.0 (0.7-1.4)	1.6 (1.2-2.3)	1.3 (0.8-1.9)
Motor composite <85	102	3.4 (2.2-5.4)	2.8 (1.8-3.9)	1.8 (1.1-3.1)	1.8 (1.1-2.7)	5.1 (3.2. 8.0)	4.1 (2.9-5.1)
**Child Behavior Checklist**							
No.	236	79	79	113	113	44	44
Internalizing T score >63	43	1.0 (0.4-2.7)	0.9 (0.3-2.4)	1.9 (0.9-3.7)	2.3 (1.1-4.5)	2.2 (0.9-5.2)	2.2 (0.6-6.4)
Externalizing T score >63	54	1.0 (0.4-2.2)	0.8 (0.3-1.8)	1.4 (0.7-2.5)	1.4 (0.7-2.7)	1.0 (0.4-2.7)	0.8 (0.2-2.7)
Total problem score >63	53	1.4 (0.6-2.9)	1.3 (0.5-2.9)	1.9 (1.1-3.4)	2.5 (1.2-4.4)	1.8 (0.8-4.2)	2.3 (0.7-5.4)

Abbreviations: aRR, adjusted relative risk; Bayley-III, Bayley Scales of Infant and Toddler Development, third edition; RR, relative risk.

^a^ Adjusted for low socioeconomic status, minority race or ethnicity, maternal primary language, no partner, Brief Symptom Inventory average, postmenstrual age at birth, infant sex, and study site.

## Discussion

Among infants born before 30 weeks’ gestation, high-risk neurobehavioral patterns prior to NICU discharge were associated with 2-year cognitive and motor delays as well as elevated behavioral problems. Dichotomization of behavioral and medical risk into low vs high was designed to evaluate outcomes among various clinical groups, with the goal of investigating whether certain profiles, specifically those that reflect poorly functioning neurobehavior, improved identification of infants at risk of 2-year delays. For this NOVI cohort, infants with high behavioral risk (NNNS profiles 5-6) but low medical risk were nearly 2 times more likely to have motor scores less than 85 on the Bayley-III and more than 2 times more likely to have internalizing and total problem T scores greater than 63 on the CBCL compared with infants with low behavioral and low medical risk. When examined in conjunction with increased medical risk, infants with high behavioral risk were nearly 3 times more likely to have low cognitive scores and 4 times more likely to have low motor scores. These results support the hypothesis that NNNS profiles enhanced the early identification of infants at risk for neurodevelopmental delay and behavior problems.

Our findings are in concert with studies that found neonatal neurobehavior associated with neurodevelopmental outcomes. In a small cohort of preterm infants, authors reported specific individual NNNS summary scores, including regulation difficulties, suboptimal reflexes, and tone abnormalities, were associated with low 18-month Bayley-III cognitive and motor scores.^12^ Clustering behavioral performance patterns into profiles provides an alternative clinical, yet comprehensive, picture of overall neurologic integrity and behavior allowing for a more holistic view. In the Maternal Lifestyle Study (MLS), Liu et al^10^ identified infants with 2 high-risk profiles, one displaying an underaroused, hypotonic pattern of behavior and the other a highly aroused, hypertonic behavioral pattern; both were associated with low gestational age and low 2-year cognitive and/or motor scores. The hyperaroused profile was also associated with elevated 3-year CBCL T scores and low 4-year IQ scores. Since profile analysis is cohort specific, it is remarkable that the 2 high-risk MLS profiles show nearly identical patterns to NOVI profiles 5 (low arousal with lethargy and hypotonia) and 6 (high arousal, high excitability, and hypertonia), thus supporting current NOVI 2-year findings and suggesting that neonatal neurobehavioral patterns displaying poor functioning can be useful in identifying childhood delays.

As expected for this very preterm cohort, rates of brain injury, necrotizing enterocolitis and/or sepsis, and BPD differed significantly between groups. BPD was the most common morbidity, ranging from 36% to 40% in low medical risk groups to 89% to 98% in high medical risk groups. BPD, sepsis, and brain injury have all been found to increase risk for early childhood motor and cognitive impairment, however with variable predictive value.^25,29,30^ Among infants with high medical risk, the addition of neurobehavioral profiles to regression models substantially increased the risk of motor scores less than 85. Additionally, significant associations of high behavioral and high medical risk with cognitive deficits were identified. Findings suggest there is a unique role for neurobehavioral assessments in the prediction of developmental outcome, as they enhance prediction of 2-year outcomes beyond identification from medical risk alone.

Risk detection efforts to identify behavioral problems among children born preterm have examined associations between NNNS neurobehavioral regulation difficulties and 2-year behavior concerns. In the 25-NICU Neonatal Adequate Care for Quality of Life Study (NEO-ACQUA), also in preterm infants, dysregulated NNNS neurobehavior reflected in excitability, stress abstinence, regulation, and handling scores predicted 18-month CBCL internalizing symptoms.^31^ NOVI findings are strikingly similar to those in NEO-ACQUA; the high behavioral risk infants had challenges with attention, regulation, and stress abstinence, and the high behavioral and low medical risk infants were 2.0 to 2.5 times more likely to have elevated CBCL internalizing and total problem scores. Interestingly, high medical risk alone was not associated with elevated CBCL scores in NOVI. This may be partly because of the low number of infants with cranial ultrasonography-identified brain injury (n = 20) and the possibility of subtle white matter injury playing a role in neurobehavior.

Early findings of neonatal dysregulation are supported by evidence linking alterations in brain structure, function, and connectivity with behavior. Injury to white matter cortical volumes and regional brain circuits have all been associated with atypical regulation, social-emotional processing, and neuromotor movements.^32,33,34,35^ Sequelae of intraventricular hemorrhage, such as ventriculomegaly and hemorrhagic infarctions, have been correlated with worse NNNS performance, including nonoptimal reflexes, tone abnormalities, and excitability.^36^ In addition, others have reported associations between brain injury, necrotizing enterocolitis, lung disease, and altered newborn behavior.^37,38^ While it is likely that there is not one pathway that disrupts neurologic and behavioral function, there is biologic plausibility to the neurobehavioral findings from the NOVI cohort that reinforce the need for continued research in the area of brain-behavior relationships.

It was not surprising that significant socioeconomic differences measured by the Hollingshead index were seen between NOVI low behavioral and high behavioral risk groups (6%-7% vs 16%-21%, respectively). The negative associations of low maternal age, education level, and IQ and poor maternal mental health with childhood behavior are well documented, yet it is suggested that these outcomes are more evident during later childhood through to adulthood.^39,40,41,42,43,44,45^ However, our results suggest that socioeconomic risk may exert clinically detectable influence early in the neonatal period, with support that epigenetic stress related mechanisms may play a role.^14^

Importantly, our NOVI findings go beyond the ability of enhancing early identification of the highest at-risk infants. A key critical component of NNNS neurobehavioral assessments is the ability to pinpoint areas of infant strengths and weaknesses, allowing clinicians to begin focusing on treatable types of behavioral difficulties prior to discharge home. The present study identified 2 unique patterns of neonatal attention, tone, movement, arousal, and stress regulation that provide an opportunity for innovative yet specifically targeted therapies. For example, caregiver-infant dyadic interventions that include social interactions, gross motor support, feeding, and routine care, would be quite different for infants with behavioral domains characterized by profile 5 vs profile 6. However, identification and initiation of such dyad-specific targeted therapies allow pediatricians and community supports (ie, Early Intervention) a potential jump start on monitoring, supporting, and providing interventions. While most studies investigating behavior problems among those born preterm take place at school age or beyond,^46,47^ research and therapeutic focus can now shift to a much earlier time period.

Broader application of NNNS assessments may be also considered. While various neurologic and behavioral tools have been used among assorted high-risk cohorts,^48,49^ the NNNS profiles specifically have evaluated performance among term infants with a variety of medical risks,^50,51^ as well as substance and toxin exposures.^10,52,53,54,55,56^ While profiles are cohort-dependent and require statistical analysis, examining patterns of readily available summary scores can allow clinicians rapid, bedside approximations of at-risk behavior, thus facilitating the neurobehavioral jump start for a variety of clinical scenarios.

### Strengths and Limitations

A unique strength of the multicenter NOVI study is the use of standardized, detailed neurobehavioral assessments to demonstrate empirical risk and help redefine how the most vulnerable infants may be identified, while at the same time informing more precise and targeted discharge planning. Innovative derivation of behavioral profiles allows for an overall clinical picture that may be used by clinicians, families, and researchers. Grouping infants into behavioral and medical risk categories allows for assessment of risk for early childhood delay beyond traditional medical risk. Furthermore, inclusion of out-born infants, as well as representation of a diverse racial and ethnic population, adds to the generalizability of finding for this heterogeneous NOVI population.

This study also has limitations, including a relatively small subgroup of high behavioral and high medical risk infants; however, despite this sample size, significant differences were observed among multiple Bayley-III and CBCL outcomes. Additionally, the follow-up rate for this study was 82%; however, the early characteristics of those without 2-year assessments were similar to those assessed. While dichotomization of risk factors and outcomes prevented a more granular, nuanced understanding of study results and implications of the NNNS assessment, it allowed for identification of clinical significance. Recognizing that 2-year outcomes may have limited school-age prediction, this cohort will continue to be followed up, allowing for exploration of longer-term outcomes and trajectories.

## Conclusions

In conclusion, among infants born less than 30 weeks’ gestation and with varying degrees of medical risk, neonatal neurobehavioral assessments at NICU discharge increased the detection of adverse cognitive and motor outcomes at 2 years of age. In addition, neurobehavioral patterns of poor regulation and functioning alone were associated with 2-year behavior problems. Thus, neonatal neurobehavioral assessments enhance the ability to identify infants at the highest risk for delays and deficits, beyond risk from severe neonatal medical conditions alone, and offer a unique, early opportunity to begin targeted therapies.

## Notes

Supplement. **eTable.** Two-Year Neurodevelopmental and Behavioral Outcomes by Medical Risk and Behavior Risk **eFigure.** NNNS Profiles

## References

[1] Kidokoro H, Anderson PJ, Doyle LW, Woodward LJ, Neil JJ, Inder TE. Brain injury and altered brain growth in preterm infants: predictors and prognosis. Pediatrics. 2014;134(2):e444-e453. doi:10.1542/peds.2013-2336 25070300

[2] Stoll BJ, Hansen NI, Bell EF, ; Eunice Kennedy Shriver National Institute of Child Health and Human Development Neonatal Research Network. Neonatal outcomes of extremely preterm infants from the NICHD Neonatal Research Network. Pediatrics. 2010;126(3):443-456. doi:10.1542/peds.2009-2959 20732945 PMC2982806

[3] Stoll BJ, Hansen NI, Bell EF, ; Eunice Kennedy Shriver National Institute of Child Health and Human Development Neonatal Research Network. Trends in care practices, morbidity, and mortality of extremely preterm neonates, 1993-2012. JAMA. 2015;314(10):1039-1051. doi:10.1001/jama.2015.10244 26348753 PMC4787615

[4] Doyle LW, Roberts G, Anderson PJ; Victorian Infant Collaborative Study Group. Outcomes at age 2 years of infants <28 weeks’ gestational age born in Victoria in 2005. J Pediatr. 2010;156(1):49-53.e1. doi:10.1016/j.jpeds.2009.07.013 19783004

[5] Van Marter LJ, Kuban KC, Allred E, ; ELGAN Study Investigators. Does bronchopulmonary dysplasia contribute to the occurrence of cerebral palsy among infants born before 28 weeks of gestation? Arch Dis Child Fetal Neonatal Ed. 2011;96(1):F20-F29. doi:10.1136/adc.2010.183012 20736416

[6] Bolisetty S, Dhawan A, Abdel-Latif M, Bajuk B, Stack J, Lui K; New South Wales and Australian Capital Territory Neonatal Intensive Care Units’ Data Collection. Intraventricular hemorrhage and neurodevelopmental outcomes in extreme preterm infants. Pediatrics. 2014;133(1):55-62. doi:10.1542/peds.2013-0372 24379238

[7] Johnson S, Marlow N. Preterm birth and childhood psychiatric disorders. Pediatr Res. 2011;69(5 Pt 2):11R-18R. doi:10.1203/PDR.0b013e318212faa0 21289534

[8] Linsell L, Johnson S, Wolke D, Morris J, Kurinczuk JJ, Marlow N. Trajectories of behavior, attention, social and emotional problems from childhood to early adulthood following extremely preterm birth: a prospective cohort study. Eur Child Adolesc Psychiatry. 2019;28(4):531-542. doi:10.1007/s00787-018-1219-8 30191335 PMC6445809

[9] Lester BM, Tronick EZ, Brazelton TB. The Neonatal Intensive Care Unit Network Neurobehavioral Scale procedures. Pediatrics. 2004;113(3 Pt 2):641-667. doi:10.1542/peds.113.S2.641 14993524

[10] Liu J, Bann C, Lester B, . Neonatal neurobehavior predicts medical and behavioral outcome. Pediatrics. 2010;125(1):e90-e98. doi:10.1542/peds.2009-0204 19969621 PMC2873896

[11] Stephens BE, Liu J, Lester B, . Neurobehavioral assessment predicts motor outcome in preterm infants. J Pediatr. 2010;156(3):366-371. doi:10.1016/j.jpeds.2009.09.042 19880137 PMC3121326

[12] El-Dib M, Massaro AN, Glass P, Aly H. Neurobehavioral assessment as a predictor of neurodevelopmental outcome in preterm infants. J Perinatol. 2012;32(4):299-303. doi:10.1038/jp.2011.100 21760584

[13] McGowan EC, Hofheimer JA, O’Shea TM, . Sociodemographic and medical influences on neurobehavioral patterns in preterm infants: a multi-center study. Early Hum Dev. 2020;142:104954. doi:10.1016/j.earlhumdev.2020.104954 32007912 PMC7115752

[14] Everson TM, Marsit CJ, Michael O’Shea T, . Epigenome-wide analysis identifies genes and pathways linked to neurobehavioral variation in preterm infants. Sci Rep. 2019;9(1):6322. doi:10.1038/s41598-019-42654-4 31004082 PMC6474865

[15] Everson TM, O’Shea TM, Burt A, . Serious neonatal morbidities are associated with differences in DNA methylation among very preterm infants. Clin Epigenetics. 2020;12(1):151. doi:10.1186/s13148-020-00942-1 33076993 PMC7574188

[16] Hofheimer JA, Smith LM, McGowan EC, . Psychosocial and medical adversity associated with neonatal neurobehavior in infants born before 30 weeks gestation. Pediatr Res. 2020;87(4):721-729. doi:10.1038/s41390-019-0607-1 31600769 PMC7082182

[17] Tronick EZ, Olson K, Rosenberg R, Bohne L, Lu J, Lester BM. Normative neurobehavioral performance of healthy infants on the Neonatal Intensive Care Unit Network Neurobehavioral Scale. Pediatrics. 2004;113(3 Pt 2):676-678. doi:10.1542/peds.113.S2.676 14993526

[18] Fink NS, Tronick E, Olson K, Lester B. Healthy newborns’ neurobehavior: norms and relations to medical and demographic factors. J Pediatr. 2012;161(6):1073-1079. doi:10.1016/j.jpeds.2012.05.036 22727876

[19] Tronick E, Lester BM. Grandchild of the NBAS: the NICU Network Neurobehavioral Scale (NNNS): a review of the research using the NNNS. J Child Adolesc Psychiatr Nurs. 2013;26(3):193-203. doi:10.1111/jcap.12042 23909942 PMC3839620

[20] Bassler D, Stoll BJ, Schmidt B, ; Trial of Indomethacin Prophylaxis in Preterms Investigators. Using a count of neonatal morbidities to predict poor outcome in extremely low birth weight infants: added role of neonatal infection. Pediatrics. 2009;123(1):313-318. doi:10.1542/peds.2008-0377 19117897 PMC2829863

[21] Bayley N. Bayley Scales of Infant Development. 3rd ed. The Psychological Corporation; 2006.

[22] Johnson S, Moore T, Marlow N. Using the Bayley-III to assess neurodevelopmental delay: which cut-off should be used? Pediatr Res. 2014;75(5):670-674. doi:10.1038/pr.2014.10 24492622

[23] Anderson PJ, Burnett A. Assessing developmental delay in early childhood—concerns with the Bayley-III scales. Clin Neuropsychol. 2017;31(2):371-381. doi:10.1080/13854046.2016.1216518 27687612

[24] Achenbach TRL. Manual for the ASEBA School-Age Forms & Profiles. University of Vermont Research Center for Children, Youth & Families; 2001.

[25] Schmidt B, Asztalos EV, Roberts RS, Robertson CM, Sauve RS, Whitfield MF; Trial of Indomethacin Prophylaxis in Preterms (TIPP) Investigators. Impact of bronchopulmonary dysplasia, brain injury, and severe retinopathy on the outcome of extremely low-birth-weight infants at 18 months: results from the trial of indomethacin prophylaxis in preterms. JAMA. 2003;289(9):1124-1129. doi:10.1001/jama.289.9.1124 12622582

[26] Zhang J, Yu KF. What’s the relative risk? a method of correcting the odds ratio in cohort studies of common outcomes. JAMA. 1998;280(19):1690-1691. doi:10.1001/jama.280.19.1690 9832001

[27] Hollingshead A. Four Factor Index of Social Status. Department of Sociology, Yale University; 1975.

[28] Deragotis LR. The Brief Symptom Inventory (BSI): Administration, Scoring and Procedures Manual. 3rd ed. National Computer Systems; 1993.

[29] Schlapbach LJ, Aebischer M, Adams M, ; Swiss Neonatal Network and Follow-Up Group. Impact of sepsis on neurodevelopmental outcome in a Swiss national cohort of extremely premature infants. Pediatrics. 2011;128(2):e348-e357. doi:10.1542/peds.2010-3338 21768312

[30] Dorner RA, Burton VJ, Allen MC, Robinson S, Soares BP. Preterm neuroimaging and neurodevelopmental outcome: a focus on intraventricular hemorrhage, post-hemorrhagic hydrocephalus, and associated brain injury. J Perinatol. 2018;38(11):1431-1443. doi:10.1038/s41372-018-0209-5 30166622 PMC6215507

[31] Montirosso R, Giusti L, De Carli P, Tronick E, Borgatti R. Developmental care, neonatal behavior and postnatal maternal depressive symptomatology predict internalizing problems at 18 months for very preterm children. J Perinatol. 2018;38(2):191-195. doi:10.1038/jp.2017.148 28933774

[32] Rogers CE, Smyser T, Smyser CD, Shimony J, Inder TE, Neil JJ. Regional white matter development in very preterm infants: perinatal predictors and early developmental outcomes. Pediatr Res. 2016;79(1-1):87-95. doi:10.1038/pr.2015.172 26372513 PMC4724306

[33] Kelly CE, Thompson DK, Cheong JL, . Brain structure and neurological and behavioural functioning in infants born preterm. Dev Med Child Neurol. 2019;61(7):820-831. doi:10.1111/dmcn.14084 30536389

[34] Rogers CE, Sylvester CM, Mintz C, . Neonatal amygdala functional connectivity at rest in healthy and preterm infants and early internalizing symptoms. J Am Acad Child Adolesc Psychiatry. 2017;56(2):157-166. doi:10.1016/j.jaac.2016.11.005 28117062 PMC5302247

[35] Eeles AL, Walsh JM, Olsen JE, . Continuum of neurobehaviour and its associations with brain MRI in infants born preterm. BMJ Paediatr Open. 2017;1(1):e000136. doi:10.1136/bmjpo-2017-000136 29637152 PMC5862173

[36] Dorner RA, Soares BP, Robinson S, Allen MC, Perin J, Burton VJ. The relationship between clinical imaging and neurobehavioral assessment in posthemorrhagic ventricular dilation of prematurity. Front Physiol. 2019;10:64. doi:10.3389/fphys.2019.00064 30804803 PMC6378306

[37] Pineda RG, Tjoeng TH, Vavasseur C, Kidokoro H, Neil JJ, Inder T. Patterns of altered neurobehavior in preterm infants within the neonatal intensive care unit. J Pediatr. 2013;162(3):470-476.e1. doi:10.1016/j.jpeds.2012.08.011 23036482 PMC3582758

[38] Brown NC, Doyle LW, Bear MJ, Inder TE. Alterations in neurobehavior at term reflect differing perinatal exposures in very preterm infants. Pediatrics. 2006;118(6):2461-2471. doi:10.1542/peds.2006-0880 17142532

[39] Peralta-Carcelen M, Carlo WA, Pappas A, ; Follow Up Committee of the Eunice Kennedy Shriver National Institute of Child Health and Human Development Neonatal Network. Behavioral problems and socioemotional competence at 18 to 22 months of extremely premature children. Pediatrics. 2017;139(6):e20161043. doi:10.1542/peds.2016-1043 28562255 PMC5470499

[40] Stoelhorst GM, Martens SE, Rijken M, ; Leiden Follow-Up Project on Prematurity. Behaviour at 2 years of age in very preterm infants (gestational age <32 weeks). Acta Paediatr. 2003;92(5):595-601. doi:10.1111/j.1651-2227.2003.tb02513.x 12839291

[41] Delobel-Ayoub M, Kaminski M, Marret S, ; EPIPAGE Study Group. Behavioral outcome at 3 years of age in very preterm infants: the EPIPAGE study. Pediatrics. 2006;117(6):1996-2005. doi:10.1542/peds.2005-2310 16740841

[42] Delobel-Ayoub M, Arnaud C, White-Koning M, ; EPIPAGE Study Group. Behavioral problems and cognitive performance at 5 years of age after very preterm birth: the EPIPAGE Study. Pediatrics. 2009;123(6):1485-1492. doi:10.1542/peds.2008-1216 19482758

[43] Potharst ES, van Wassenaer AG, Houtzager BA, van Hus JW, Last BF, Kok JH. High incidence of multi-domain disabilities in very preterm children at five years of age. J Pediatr. 2011;159(1):79-85. doi:10.1016/j.jpeds.2010.12.055 21349538

[44] Hoffman L, Bann C, Higgins R, Vohr B; Eunice Kennedy Shriver National Institute of Child Health and Human Development Neonatal Research Network. Developmental outcomes of extremely preterm infants born to adolescent mothers. Pediatrics. 2015;135(6):1082-1092. doi:10.1542/peds.2014-3880 25963007 PMC4444804

[45] Evans GW, Li D, Whipple SS. Cumulative risk and child development. Psychol Bull. 2013;139(6):1342-1396. doi:10.1037/a0031808 23566018

[46] Arpi E, Ferrari F. Preterm birth and behaviour problems in infants and preschool-age children: a review of the recent literature. Dev Med Child Neurol. 2013;55(9):788-796. doi:10.1111/dmcn.12142 23521214

[47] Duncan AF, Matthews MA. Neurodevelopmental outcomes in early childhood. Clin Perinatol. 2018;45(3):377-392. doi:10.1016/j.clp.2018.05.001 30144844

[48] Romeo DM, Cowan FM, Haataja L, . Hammersmith Infant Neurological Examination in infants born at term: predicting outcomes other than cerebral palsy. Dev Med Child Neurol. 2022;64(7):871-880. doi:10.1111/dmcn.15191 35201619

[49] Robinson H, Hart D, Vollmer B. Predictive validity of a qualitative and quantitative Prechtl’s General Movements Assessment at term age: comparison between preterm infants and term infants with HIE. Early Hum Dev. 2021;161:105449. doi:10.1016/j.earlhumdev.2021.105449 34481188

[50] Parikh AN, Triplett RL, Wu TJ, . NICU Network Neurobehavioral Scale profiles in term infants: associations with maternal adversity, medical risk, and neonatal outcomes. J Pediatr. Published online April 14, 2022. doi:10.1016/j.jpeds.2022.04.016 PMC10030163 35430247

[51] Hogan WJ, Winter S, Pinto NM, . Neurobehavioral evaluation of neonates with congenital heart disease: a cohort study. Dev Med Child Neurol. 2018;60(12):1225-1231. doi:10.1111/dmcn.13912 29748956

[52] Wouldes TA, Woodward LJ. Neurobehavior of newborn infants exposed prenatally to methadone and identification of a neurobehavioral profile linked to poorer neurodevelopmental outcomes at age 24 months. PLoS One. 2020;15(10):e0240905. doi:10.1371/journal.pone.0240905 33064777 PMC7567379

[53] Appleton AA, Murphy MA, Koestler DC, . Prenatal programming of infant neurobehaviour in a healthy population. Paediatr Perinat Epidemiol. 2016;30(4):367-375. doi:10.1111/ppe.12294 27004434 PMC5054721

[54] Tung PW, Burt A, Karagas M, . Association between placental toxic metal exposure and NICU Network Neurobehavioral Scales (NNNS) profiles in the Rhode Island Child Health Study (RICHS). Environ Res. 2022;204(Pt A):111939. doi:10.1016/j.envres.2021.111939 34461121 PMC8639656

[55] Flannery T, Davis JM, Czynski AJ, . Neonatal abstinence syndrome severity index predicts 18-month neurodevelopmental outcome in neonates randomized to morphine or methadone. J Pediatr. 2020;227:101-107.e1. doi:10.1016/j.jpeds.2020.08.034 32805259 PMC7731918

[56] Zhang X, Spear E, Gennings C, . The association of prenatal exposure to intensive traffic with early preterm infant neurobehavioral development as reflected by the NICU Network Neurobehavioral Scale (NNNS). Environ Res. 2020;183:109204. doi:10.1016/j.envres.2020.109204 32311904 PMC7325861

