# Supplementary material for: Analysis of Neonatal Neurobehavior and Developmental Outcomes Among Preterm Infants
Source: JAMA Netw Open. 2022 Jul 18;5(7):e2222249. doi: 10.1001/jamanetworkopen.2022.22249 (PMC9294999; doi:10.1001/jamanetworkopen.2022.22249)
Supplement: Supplement. — eTable. Two-Year Neurodevelopmental and Behavioral Outcomes by Medical Risk and Behavior Risk eFigure. NNNS Profiles [file jamanetwopen-e2222249-s001.pdf]

# Supplemental Online Content

McGowan EC, Hofheimer JA, O'Shea TM, et al. Analysis of neonatal neurobehavior and developmental outcomes among preterm infants. *JAMA Netw Open.* 2022;5(7):e2222249. doi:10.1001/jamanetworkopen.2022.22249

**eTable.** Two-Year Neurodevelopmental and Behavioral Outcomes by Medical Risk and Behavior Risk

**eFigure.** NNNS Profiles

This supplemental material has been provided by the authors to give readers additional information about their work.

© 2022 McGowan EC et al. *JAMA Network Open.*

| **eTable.** Two-Year Neurodevelopmental and Behavioral Outcomes by Medical Risk* and Behavior Risk** | | | | |
|---|---|---|---|---|
| **Outcome** | **Low Behavior Risk + Low Medical Risk** | **Low Behavior Risk + High Medical Risk** | **High Behavior Risk + Low Medical Risk** | **High Behavior Risk + High Medical Risk** |
| **Bayley-III[a]** | **n=299** | **n=76** | **n=106** | **n=38** |
| Cognitive composite < 85 | 60 (20.1%) | 22 (28.9%) | 24 (22.6%) | 20 (52.6%) |
| Language composite < 85 | 101 (33.8%) | 37 (48.7%) | 40 (37.7%) | 21 (55.3%) |
| Motor composite < 85 | 31 (10.4% ) | 27 (35.5%) | 20 (18.9%) | 20 (52.6%) |
| | | | | |
| **Child Behavior Checklist (CBCL)[a]** | **n=308** | **n=79** | **n=113** | **n=44** |
| Internalizing T-Score >63 | 19 (6.2%) | 5 (6.3%) | 13 (11.5%) | 6 (13.6%) |
| Externalizing T-Score >63 | 28 (9.1%) | 7 (8.9%) | 14 (12.4% ) | 4 (9.1%) |
| Total Problem Score >63 | 23 (7.5%) | 8 (10.1%) | 16 (14.2%) | 6 (13.6%) |

* Medical risks include the following neonatal morbidities: chronic lung disease, brain injury (parenchymal echodensity, periventricular leukomalacia, ventricular dilation), necrotizing enterocolitis/sepsis, severe retinopathy of prematurity. **Low medical risk** defined as ≤ 1 morbidity, **high medical risk** defined as ≥ 2 morbidities.

** Behavioral risk grouped by NNNS profiles: **low behavioral risk** defined as Profiles 1-4, **high behavioral risk** defined as Profile 5-6

© 2022 McGowan EC et al. *JAMA Network Open.*

**eFigure. NNNS Profiles**

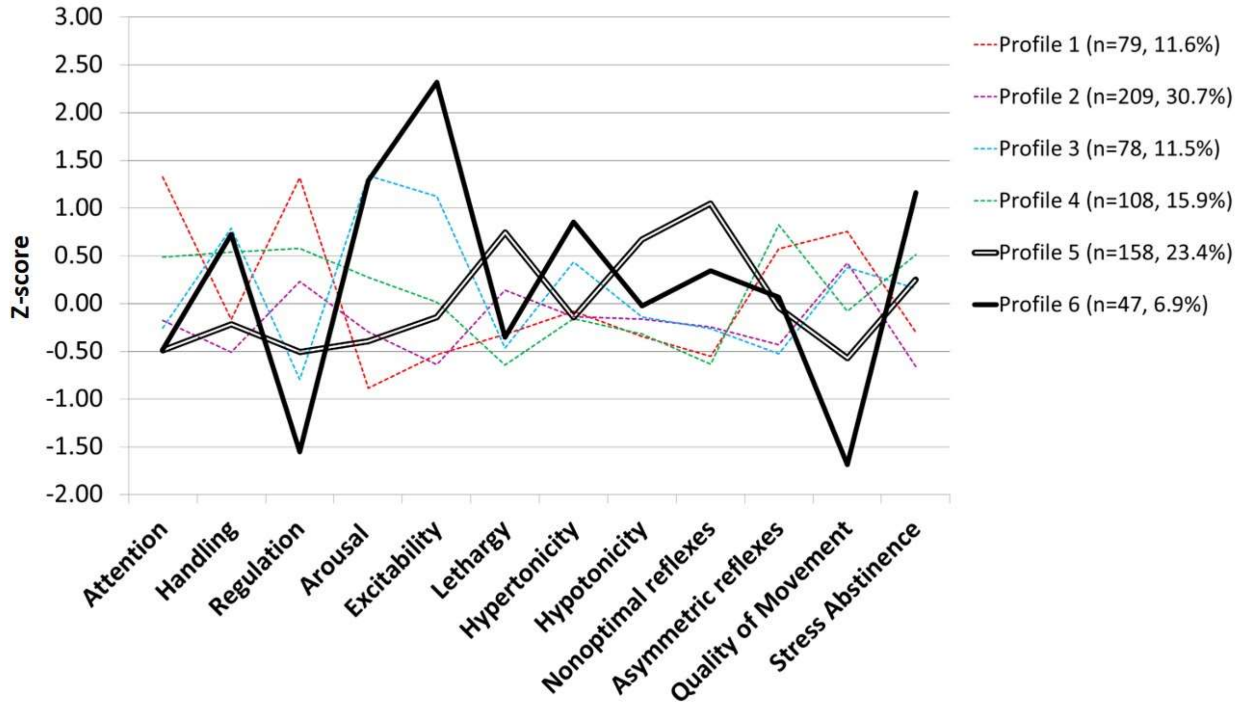


**Profiles 5 & 6 high risk behavioral profiles*

© 2022 McGowan EC et al. *JAMA Network Open.*
